# Correction: An international consensus on effective, inclusive, and career-spanning short-format training in the life sciences and beyond

**DOI:** 10.1371/journal.pone.0319613

**Published:** 2025-02-20

**Authors:** Jason J. Williams, Rochelle E. Tractenberg, Bérénice Batut, Erin A. Becker, Anne M. Brown, Melissa L. Burke, Ben Busby, Nisha K. Cooch, Allissa A. Dillman, Samuel S. Donovan, Maria A. Doyle, Celia W. G. van Gelder, Christina R. Hall, Kate L. Hertweck, Kari L. Jordan, John R. Jungck, Ainsley R. Latour, Jessica M. Lindvall, Marta Lloret-Llinares, Gary S. McDowell, Rana Morris, Teresa Mourad, Amy Nisselle, Patricia Ordóñez, Lisanna Paladin, Patricia M. Palagi, Mahadeo A. Sukhai, Tracy K. Teal, Louise Woodley

There are errors in the Funding statement. The correct Funding statement is as follows: This work was supported by the National Science Foundation under DRL/EHR: 2027025 to JJW & RET. This funder played no role in the study design, data collection and analysis, decision to publish, or preparation of the manuscript. Any opinions, findings, and conclusions or recommendations expressed in this material are those of the author(s) and do not necessarily reflect the views of the National Science Foundation. Several co-authors are employed by commercial entities. These employers provided support in the form of salaries for BB, KLJ, EAB, and NC and did not have any additional role in the study design, data collection and analysis, decision to publish, or preparation of the manuscript. The specific roles of these authors are articulated in the ‘author contributions’ section

There are errors in the Competing interests. The correct Competing interests is as follows: This work was supported by the National Science Foundation under DRL/EHR: 2027025 to JJW & RET. This funder played no role in the study design, data collection and analysis, decision to publish, or preparation of the manuscript. Any opinions, findings, and conclusions or recommendations expressed in this material are those of the author(s) and do not necessarily reflect the views of the National Science Foundation. Several co-authors are employed by commercial entities. These employers provided support in the form of salaries for BB, KLJ, EAB, and NC and did not have any additional role in the study design, data collection and analysis, decision to publish, or preparation of the manuscript. The specific roles of these authors are articulated in the ‘author contributions’ section
