# Supplementary material for: An international consensus on effective, inclusive, and career-spanning short-format training in the life sciences and beyond
Source: PLoS One. 2023 Nov 9;18(11):e0293879. doi: 10.1371/journal.pone.0293879 (PMC10635508; doi:10.1371/journal.pone.0293879)
Supplement: S1 Text — List of definitions used and agreed upon by the authors. (DOCX) [file pone.0293879.s001.docx]

**SUPPLEMENTAL INFORMATION: Williams, Tractenberg et al., "An International Consensus on Effective, Inclusive, and Career-spanning Short-format Training in the Life Sciences and Beyond "**

**S1. Short-Format Training (SFT) definition**

*This was the full definition of SFT developed and used by participants at the workshop.*

Short-format training involves instruction in disciplinary skills and knowledge over a relatively short duration (i.e., hours, days, or a few weeks). Rather than specifying a set number of hours, the easiest way to identify SFT is that it will be labeled as a workshop, bootcamp, short-course, or similar term. SFT generally has the following features:

- Generally happens outside of a formal undergraduate, graduate, or other degree-granting program. Generally, SFT learners do not receive a summative grade or university course credits. SFT may also include post-degree training programs for professional accreditation (e.g., physicians or medical specialists training for fellowship exams in a specialty). Learners may be recognized as having earned “professional development” or “continuing education” credit.
- Content is determined by instructors or instructional designers, not necessarily by an institutional or professional society curriculum approval process (e.g., accreditation).
- Tends to be stand-alone, not requiring formal prerequisites or required subsequent SFT. There may be expectations for learner preparation or recommended prerequisite knowledge. SFT can be linked and can have prerequisites, but these characteristics are not enforced as they might be in a formal setting.
- Typically delivered to a group of learners who enroll because of their interest in the topic, rather than a mandate.
- Typically developed and delivered by domain experts outside of and separately from an institutional undergraduate/graduate coursework teaching role (if they have such a role).

The extent to which a specific SFT course meets any of the above features will vary.
