# Supplementary material for: An international consensus on effective, inclusive, and career-spanning short-format training in the life sciences and beyond
Source: PLoS One. 2023 Nov 9;18(11):e0293879. doi: 10.1371/journal.pone.0293879 (PMC10635508; doi:10.1371/journal.pone.0293879)
Supplement: S2 Text — Details on how call for participation was distributed. (DOCX) [file pone.0293879.s002.docx]

**SUPPLEMENTAL INFORMATION: Williams, Tractenberg et al., "An International Consensus on Effective, Inclusive, and Career-spanning Short-format Training in the Life Sciences and Beyond "**

**S2. Community Distribution**

**Communities and Mailing Lists from which nominations were invited**

The conference and nomination process was circulated to the following groups and lists: The Carpentries (>3,000 volunteer instructors), CyVerse mailing list (>80,000 subscribers), ELIXIR, Australian BioCommons (>1,500 subscribers), and Galaxy Training Networks; NIH intramural training and extramural research offices, LifeSciTrainers.org blog, Open Life Science; the Global Organisation for Bioinformatics Learning, Education and Training (GOBLET); BioQUEST Curriculum Consortium (20,000 users and >6,000 subscribers); through the Co-PIs of the NSF RCN-UBE: Establishing a Genomics Education Alliance: Steps toward Sustainability (GEA) and affiliated networks like the Genomics Educational Partnership (GEP).
