# Supplementary material for: An international consensus on effective, inclusive, and career-spanning short-format training in the life sciences and beyond
Source: PLoS One. 2023 Nov 9;18(11):e0293879. doi: 10.1371/journal.pone.0293879 (PMC10635508; doi:10.1371/journal.pone.0293879)
Supplement: S3 Text — Initial problem set considered by participants. (DOCX) [file pone.0293879.s003.docx]

**SUPPLEMENTAL INFORMATION: Williams, Tractenberg et al., "An International Consensus on Effective, Inclusive, and Career-spanning Short-format Training in the Life Sciences and Beyond "**

**S3. Draft Challenge Vignette List for Kick-Off meeting.**

Based upon our original proposal, consultation with the organizing committee, and discussion with community members, we identified an initial problem set that could potentially be addressed at the conference (submitted to all participants ahead of kick-off meeting):

1. How can an instructor ensure that everyone who needs to know, knows about a learning

opportunity?

1. People expect to be given high quality training for free, and

simultaneously consider free to mean low quality; how can instructors resolve this conflicting “truth” about online training?

1. How can I integrate ethical content into my course?
2. How do instructors establish, gauge, and maintain learners’ engagement?
3. How do instructors deliver content when the subject matter is in

flux?

1. How do instructors begin developing a course when there don’t

seem to be pre-existing training materials on a topic?

1. How should SFT be structured so that learners who misinterpret or fail to meet

prerequisites still have a good chance of a successful learning experience?

1. Can learners be helped to differentiate “good” from “bad”

instruction before their SFT experience?

1. How can instructors know if their assessments are working?
2. How can instructors assess catalysis? Is it enough to add it, or do

you have to assess it?

1. How could instructors advise learners with highly individualized needs about future

learning? How important is it that instructors identify individuals with unique needs?

1. How might instructors know if their learners are succeeding *after*

an SFT event?

1. How can learners determine if an SFT is appropriate for them?
2. How should instructors plan for diverse needs of learners?
3. How should instructors manage the learning environment to

maximize participation, and identify and minimize barriers to engagement?

1. How can we facilitate learners to define their specific needs in SFT?
2. What should instructors do if they don’t have time to design,

deliver, and/or grade assessments?

1. What would make training materials more reusable for

instructors? At what point in development should this be considered?

1. What are incentives to adopt/disincentives from adopting specific

(evidence-based) teaching practices?

1. How could instructors increase the diversity of attendees who

participate in training?
