# Supplementary material for: An international consensus on effective, inclusive, and career-spanning short-format training in the life sciences and beyond
Source: PLoS One. 2023 Nov 9;18(11):e0293879. doi: 10.1371/journal.pone.0293879 (PMC10635508; doi:10.1371/journal.pone.0293879)
Supplement: S4 Text — Framing document used to orient participants to this project and its goals. (DOCX) [file pone.0293879.s004.docx]

**SUPPLEMENTAL INFORMATION: Williams, Tractenberg et al., "An International Consensus on Effective, Inclusive, and Career-spanning Short-format Training in the Life Sciences and Beyond "**

**S4. *Precis***

A Set of Principles for Making Career-spanning Learning in the Life Sciences Inclusive and Effective for All

The rapidly-increasing interdisciplinarity of the life sciences makes career-spanning learning critical. Scientists who are able to recognize and traverse skills gaps are better positioned to pursue impactful science and achieve their personal career goals. To bolster their skills, life scientists often turn to short-format training (e.g., workshops, bootcamps, and short courses) for professional development. While short-format training (SFT) provides point-of-need help, and is often low in time commitment and costs, this approach can be less successful than typically assumed.

We propose a set of principles for ensuring that short-format training in the life sciences are effective and accessible to all. “The principles” are intended to promote the professionalization of SFT and can support several aspirational outcomes:

1. Enable stakeholders to design (instructors), select (learners), or deploy (institutions, communities, and funders) effective, catalytic, and inclusive training opportunities in support of career-spanning learning.

2. Empower learners to reliably assess if a short-format professional development opportunity will help them achieve their learning goals by promoting the adoption of evidence-based practices from the learning sciences.

3. Establish inclusivity and “catalysis” (support for ongoing self-directed learning) as essential to short-format training.

Buy-in and application of these principles for SFT could influence how we approach career-spanning learning in the sciences, supporting our vision of career-spanning training that is inclusive and effective for all.

**The principles, challenges, and work of the conference**

We describe each of the principles below with rationale and example questions that might arise in the design, planning, delivery, and refinement of SFT. We attempt to show that the principles we have identified accommodate a diverse set of challenges and ties to our purpose of advancing inclusive and effective career-spanning learning.

We also have listed potential challenges that we can address at the conference. The challenges (which include your suggestions and feedback) are meant to illustrate realistic problems SFT learners and instructors face. The set we’ve selected is open to expansion and refinement (see “evaluating the principles” at the conclusion of this document). During the December kickoff, we will focus on challenges that are directly within the purview of instructors to address. We also realize broader challenges exist; we should document them as we identify them and will have a separate approach for working on these at the May conference.

During kickoff, small groups will work on the challenges that resonate most with them; we don’t anticipate addressing all possible challenges. An accompanying “worksheet” will present a structured process for each small group to refine that challenge, adapt the challenge (if needed) to a specific setting, and propose structured solutions that might address that challenge. The worksheet will prompt you to consider specific lines of evidence including drawing on the education research literature (see: Workshop Reading) as well as your own expertise and experience. Small-group discussions, evaluation against the principles, reformulations of groups, etc. will hopefully lead to strategies and recommendations that will resonate with others.

**CORE PRINCIPLES I-IV**

The first set of principles (Best Evidence, Effective, Catalytic, and Inclusive) are referred to as “core” because we believe all SFT must address them. Numbering of the principles suggests a sequence (e.g. training needs to be effective before trying to maximize its inclusivity), but the temporal order is not necessarily strict and all are required.

Designing SFT based on best available evidence from the learning sciences (peer-reviewed literature, books, and experience) is the first principle since the creation of learning objectives is universally agreed to be the optimal starting place for effective teaching. Once lessons are created, evidence of their effectiveness - based on assessment that is aligned with the objectives and the instruction - is the second principle. Determining if SFT supports sustainable/ongoing, self-directed learning, which we have designated “catalysis”, is a novel component to establishing effectiveness. Because SFT is typically intended to augment the life scientist-learner’s skill set, and not just their foundational knowledge, we characterize instruction as effective if it both supports the learners’ achievement of learning objectives but also supports ongoing learning and application of the new knowledge - i.e., if SFT is catalytic, then it serves its ostensible purpose. Thus, catalysis is the third principle, expanding what we consider “effective” instruction beyond achieving the more immediately verifiable learning objectives. We assert that training which cannot promote catalysis for learners is not fully effective. Inclusivity is the fourth principle, not because it is less important than others but because we believe disseminating ineffective training, however inclusive, is unhelpful and harmful. Some elements of inclusivity are applicable at the best evidence phase of instructional design, whereas different inclusivity features may be relevant for determining SFT effectiveness or catalysis. Finally, inclusivity requires ongoing refinements that may become apparent as more learners provide feedback over time.

Bicycle Principles:

**I. Best Evidence**

SFT must be grounded in evidence-based practices from educational research.

**II/III. Effective and Catalytic**

SFT should be effective, providing evidence to learners that they have made progress in achieving learning goals. Learners should be assisted in formulating additional learning goals and prepared for future self-directed learning.

**COMMUNITY PRINCIPLES V-VII**

The second set of features (Shareable, Scalable, and Sustainable) are called community principles because we posit that these emerge when groups and communities of individuals think about long-term impacts of training and the potential to build skills for large groups of people. Developers of SFT may have no intention of making their training broadly available by sharing (increasing the numbers of instructors) or scaling (increasing the number of learners). However, when training is shared/scaled. These principles could be used to promote organized progress across the discipline.

**V/VI/VII. Shareable, Scalable, and Sustainability with Fidelity**

Training that meets the Core Principles can be shared with new and larger audiences. This sharing should be done in a way that maximizes fidelity to the Core Principles even when adaptations are made.

**CONCLUSION**

Evaluating the Principles

These Principles are a starting point for the conference, and will hopefully provide a common point of focus for us to exchange ideas around. Are they complete? Can you think of any important principles that are missing or don’t fit? Are these principles potentially a starting point for SFT instructors everyone to coalesce around or buy in to? We must also consider the specific conditions of the life sciences and how these principles may apply in this and other scientific domains.
