# Supplementary material for: An international consensus on effective, inclusive, and career-spanning short-format training in the life sciences and beyond
Source: PLoS One. 2023 Nov 9;18(11):e0293879. doi: 10.1371/journal.pone.0293879 (PMC10635508; doi:10.1371/journal.pone.0293879)
Supplement: S5 Text — Detailed example of a recommendation produced by the author group. (DOCX) [file pone.0293879.s005.docx]

**SUPPLEMENTAL INFORMATION: Williams, Tractenberg et al., "An International Consensus on Effective, Inclusive, and Career-spanning Short-format Training in the Life Sciences and Beyond "**

**S5. Complete Example: Recommendation A.**

Each of the 14 recommendations has several descriptive features:

- **Summary**: Expands upon the problem(s) the recommendation tries to solve.
- **How might this work**: Brief implementation example and suggestions on evaluating success.
- **Related Principles**: Most closely related Bicycle Principles.
- **Benefits to the learners**: How recommendation helps learners (directly or indirectly).
- **Incentives to Implementers**: Motivations for implementers to enact this recommendation.
- **Barriers to Implementation**: Obstacles that may hinder this recommendation.

We provide here an example of one full recommendation as developed by conference participants. The complete list of recommendations and descriptions are available at www.bikeprinciples.org. Please note this website also contains additional detailed definitions of terms

(see: https://www.bikeprinciples.org/#definitions/)

**A. Professionalize the training of short-format training instructors and instructional designers**

**Summary:** Creating a professional society(ies) or communities of practice dedicated to SFT could support instructors, instructional designers, and instructional administrators, as well as help to extend the reach of the *Bicycle Principles*.

Instructors, instructional designers, and instructional supporters/administrators could benefit from services provided by professional societies (e.g., a venue for self-identification, shared affiliation; dissemination of work; development of practice; rewarding of outstanding contributions). Instead of being a “special interest group” splintered from a specific professional society, this could be an independent, cross-cutting, professional community that can promote appreciation for the professional competency of effective SFT instructors. It could also positively influence the entire scientific community by promoting effective and accessible training principles worldwide. This group could focus on training to assist SFT instructors and instructional designers to reach specific standards. Since most professional associations already have instruction-focused special interest groups, there is evidence many people would be interested. Furthermore, existing but unconnected groups around the world already have formal, iteratively-improving trainer-training programs in which new instructors engage.

**How might this work:**

A SFT training professional society could be jointly established by organizations and/or projects that have significant SFT interest, expertise, and activity. As a community of practice, the society could: 1) vet, curate, and/or maintain centralized resources; 2) work to clarify and address significant challenges; and 3) share innovations for the delivery of effective, inclusive, and career-spanning SFT. Like most professional societies there could be virtual and in-person convenings, topical or geographical sections, and various communication mechanisms. Participation in this community would support recognition of peers and the strengthening and promotion of SFT teaching competencies.

Success for this new professional society would look like a growing membership base and the co-production of actionable artifacts for others to reuse (e.g., recommendations, guidelines, methods). The unique mission of this society is its emphasis on improving the consistency and quality of SFT training, at the level of the individual instructor and instructional designers as a profession. Individuals from diverse scientific disciplines could join this society to signal their commitment to effective, inclusive SFT instruction.

**Related Principles:**

- All

**Benefits to the learners:**

1. Increases the likelihood of successful SFT learning experiences when instructors have benefited from training and knowledge exchange promoted by the SFT professional society.
2. More high-quality instructors trained by the society increase the chance of meeting learner demand for SFT.

**Incentives to implementers:**

*For Instructors*

1. Recognition of their role and its importance in the scientific community, peer support, and increased sense of community. Professionalization may counter the perception that SFT (which is often delivered at no cost to learners) is not of “real value”.

*For Instructors, Instructional Designers, Instructional Supporters*

1. Collective impact could disseminate effective practices and enhance the adoption of practices that individual instructors or SFT programs might have difficulty implementing alone.
2. Opportunities to develop new, better, and sustainable economic models for SFT as well as models for applying community *Bicycle Principles* (i.e., *Reach*, *Scale*, *Sustain*).

*For Funders and Organizations*

1. Working with an SFT professional society may strengthen justifications for SFT funding and allow funders to interact with multiple programs through a single group. The professionalization of instructors brings value to SFT provided by organizations.

**Barriers to implementation:**

1. There must be sufficient global grassroots support to form such an organization, and this requires clear value propositions for members and financial sponsors.
2. There are legal, logistical, and financial barriers to launching an entirely new organization. It may be possible for existing organizations and nonprofits to help builders leverage existing resources and groups.
3. To be successful, this organization will have to demonstrate significant and positive effects on the community of instructors and instruction developers. Many of these community members offer SFT as simply a transient or minor activity. If the new organization can show benefits for them, it will strengthen the effort.
