# Supplementary material for: An international consensus on effective, inclusive, and career-spanning short-format training in the life sciences and beyond
Source: PLoS One. 2023 Nov 9;18(11):e0293879. doi: 10.1371/journal.pone.0293879 (PMC10635508; doi:10.1371/journal.pone.0293879)
Supplement: S6 Text — A list of all recommendations developed and accepted by the authoring group. (DOCX) [file pone.0293879.s006.docx]

**SUPPLEMENTAL INFORMATION: Williams, Tractenberg et al., "An International Consensus on Effective, Inclusive, and Career-spanning Short-format Training in the Life Sciences and Beyond "**

**S6. Full list of 14 Recommendations**

A. Professionalize the training of short-format training instructors and instructional designers.

B. Centralize infrastructure for short-format training assessment and evaluation.

C. Support microcredentialing of short-format training instructors.

D. Operationalize equitable and inclusive practice in short-format training as an ethical obligation.

E. Make the Bicycle Principles actionable for funders.

F. Deploy short-format training to counter inequity.

G. Clarify the economic models that enable short-format training.

H. Document models for high-fidelity reaching, scaling, and/or sustaining of short-format training.

I. Encourage interoperable short-format training registries.

J. Apply FAIR principles to training materials.

K. Communicate standards of instruction through badging.

L. Develop an implementation strategy for Catalytic learning.

M. Support integration of diagnostic assessment into short-format training.

N. Encourage evidence-based guidance to support career-spanning learning.
