# Supplementary material for: An international consensus on effective, inclusive, and career-spanning short-format training in the life sciences and beyond
Source: PLoS One. 2023 Nov 9;18(11):e0293879. doi: 10.1371/journal.pone.0293879 (PMC10635508; doi:10.1371/journal.pone.0293879)
Supplement: Attachment — Submitted filename: plos1.14 recommendations.Responses to Reviewers.12oct.docx [file pone.0293879.s007.docx]

**Responses to Reviewers: PONE-D-23-21707: An International Consensus on Effective, Inclusive, and Career-spanning Short-format Training in the Life Sciences and Beyond**

***Data availability***

We have clarified that all appropriate data, in the form of the starting cases, three ultimate focus points, and recommendations are contained within the manuscript. We have also clarified that the intermediate products of the conference were kept confidential to ensure free and full engagement and contributions from all participants. Delphi best practices - now referenced in the text - specify that engagement by experts should be anonymized, to ensure free and open contributions. Thus, the intermediate outputs (data) are neither appropriate nor approved to share.

***Amend the abstract to briefly mention the delphi method and the participant demographics.***

We've listed their number (30) and countries (10), and briefly mentioned the live Delphi in the abstract.

Please submit your revised manuscript by Oct 19 2023 11:59PM. If you will need more time than this to complete your revisions, please reply to this message or contact the journal office at [plosone@plos.org](mailto:plosone@plos.org). When you're ready to submit your revision, log on to <https://www.editorialmanager.com/pone/> and select the 'Submissions Needing Revision' folder to locate your manuscript file.

Please include the following items when submitting your revised manuscript:

- A rebuttal letter that responds to each point raised by the academic editor and reviewer(s). You should upload this letter as a separate file labeled 'Response to Reviewers'.
- A marked-up copy of your manuscript that highlights changes made to the original version. You should upload this as a separate file labeled 'Revised Manuscript with Track Changes'.
- An unmarked version of your revised paper without tracked changes. You should upload this as a separate file labeled 'Manuscript'.

If you would like to make changes to your financial disclosure, please include your updated statement in your cover letter. Guidelines for resubmitting your figure files are available below the reviewer comments at the end of this letter.

If applicable, we recommend that you deposit your laboratory protocols in [protocols.io](http://protocols.io/) to enhance the reproducibility of your results. Protocols.io assigns your protocol its own identifier (DOI) so that it can be cited independently in the future. For instructions see: <https://journals.plos.org/plosone/s/submission-guidelines#loc-laboratory-protocols>. Additionally, PLOS ONE offers an option for publishing peer-reviewed Lab Protocol articles, which describe protocols hosted on [protocols.io](http://protocols.io/). Read more information on sharing protocols at <https://plos.org/protocols?utm_medium=editorial-email&utm_source=authorletters&utm_campaign=protocols>.

Journal Requirements:

When submitting your revision, we need you to address these additional requirements.

1. Please ensure that your manuscript meets PLOS ONE's style requirements, including those for file naming. The PLOS ONE style templates can be found at 
<https://journals.plos.org/plosone/s/file?id=wjVg/PLOSOne_formatting_sample_main_body.pdf> and 
<https://journals.plos.org/plosone/s/file?id=ba62/PLOSOne_formatting_sample_title_authors_affiliations.pdf>

2. Thank you for stating the following in the Acknowledgments Section of your manuscript: 
   "This material is based upon work supported by the National Science Foundation under DRL/EHR:2027025. Any opinions, findings, and conclusions or recommendations expressed in this material are those of the author(s) and do not necessarily reflect the views of the National Science Foundation. The authors wish to thank Rebecca Leshan and the staff of the CSHL Banbury Center for their guidance and facilitation of our convenings, and other participants in the conference including Charla Lambert."

*We note that you have provided funding information that is not currently declared in your Funding Statement. However, funding information should not appear in the Acknowledgments section or other areas of your manuscript. We will only publish funding information present in the Funding Statement section of the online submission form. 
Please remove any funding-related text from the manuscript and let us know how you would like to update your Funding Statement.*

line 281 - removed the grant #.

Currently, your Funding Statement reads as follows: 
  "This work was supported by the National Science Foundation under DRL/EHR: 2027025 to JWW & RET. Funders played no role in the study design, data collection and analysis, decision to publish, or preparation of the manuscript."

*Please include your amended statements within your cover letter; we will change the online submission form on your behalf.*

line 281 - removed the grant #. We do not need to update the funding statement, as the US NSF was the only funder for the conference. We did add " Any opinions, findings, and conclusions or recommendations expressed in this material are those of the author(s) and do not necessarily reflect the views of the National Science Foundation." to the funding statement.

*3. Thank you for stating the following in the Competing Interests section: 
   "B.B.: I have read the journal's policy and  have the following competing interest: I am an employee of DNAnexus.
G.S.M.: I have read the journal's policy and have the following competing interest: I work as an independent consultant who provides short-format training and professional development services. 
K.L.J.: I have read the journal's policy and have the following competing interest: I am* *an employee of The Carpentries.
E.A.B.: I have read the journal's policy and have the following competing interest: I am an employee of The Carpentries. 
N.C.: I have read the journal's policy and have the following competing interest: I am the owner of CureComms Advisors LLC and was compensated for participating in the meeting and providing strategic content guidance. 
No other authors declare competing interests."*

*We note that one or more of the authors are employed by a commercial company: DNAnexus and The Carpentries.*

*a. Please provide an amended Funding Statement declaring this commercial affiliation, as well as a statement regarding the Role of Funders in your study. If the funding organization did not play a role in the study design, data collection and analysis, decision to publish, or preparation of the manuscript and only provided financial support in the form of authors' salaries and/or research materials, please review your statements relating to the author contributions, and ensure you have specifically and accurately indicated the role(s) that these authors had in your study. You can update author roles in the Author Contributions section of the online submission form.*

Participation by employees of these companies was not funded by the companies; their employers did not contribute any funding to the project. All author contributions are correct as originally stated (no changes).

*Please also include the following statement within your amended Funding Statement. 
“The funder provided support in the form of salaries for authors [insert relevant initials], but did not have any additional role in the study design, data collection and analysis, decision to publish, or preparation of the manuscript. The specific roles of these authors are articulated in the ‘author contributions’ section.”
If your commercial affiliation did play a role in your study, please state and explain this role within your updated Funding Statement.*

We have added the specified text, “The funder provided support in the form of salaries for authors BB, KLJ, EAB, and NC, but did not have any additional role in the study design, data collection and analysis, decision to publish, or preparation of the manuscript. The specific roles of these authors are articulated in the ‘author contributions’ section.” to the cover letter.

*b. Please also provide an updated Competing Interests Statement declaring this commercial affiliation along with any other relevant declarations relating to employment, consultancy, patents, products in development, or marketed products, etc.*

*Within your Competing Interests Statement, please confirm that this commercial affiliation does not alter your adherence to all PLOS ONE policies on sharing data and materials by including the following statement: "This does not alter our adherence to  PLOS ONE policies on sharing data and materials.” (as detailed online in our guide for authors* [*http://journals.plos.org/plosone/s/competing-interests*](http://journals.plos.org/plosone/s/competing-interests)*) . If this adherence statement is not accurate and  there are restrictions on sharing of data and/or materials, please state these. Please note that we cannot proceed with consideration of your article until this information has been declared.*

*Please include both an updated Funding Statement and Competing Interests Statement in your cover letter. We will change the online submission form on your behalf.*

We have added the specified text to the cover letter.

*4. We noted in your submission details that a portion of your manuscript may have been presented or published elsewhere. [The manuscript was submitted to biorxiv in march 2023. The submitted version is a more concise version (same results), and is formatted for PLOS one. Williams J, Tractenberg RE, Batut B, Becker E, Brown A, Burke M, et al. (2023, 15 March). Optimizing Short-format Training: an International Consensus on Effective, Inclusive, and Career-spanning Professional Development in the Life Sciences and Beyond. bioRxiv 2023.03.10.531570; doi:* [*https://doi.org/10.1101/2023.03.10.531570*](https://doi.org/10.1101/2023.03.10.531570)*] Please clarify whether this [conference proceeding or publication] was peer-reviewed and formally published. If this work was previously peer-reviewed and published, in the cover letter please provide the reason that this work does not constitute dual publication and should be included in the current manuscript.*

This has been clarified.

*5. Please include captions for your Supporting Information files at the end of your manuscript, and update any in-text citations to match accordingly. Please see our Supporting Information guidelines for more information:* [*http://journals.plos.org/plosone/s/supporting-information*](http://journals.plos.org/plosone/s/supporting-information)*.*

6. Please review your reference list to ensure that it is complete and correct.

**Comments to the Author**

5. Review Comments to the Author

***Reviewer #1: About “statistical analysis”: I selected “I don’t know” because the authors use content analysis, but don’t discuss much about what they did.***

Lines 274-280 and 296-317 now detail the Delphi and content analysis methods.

***About data availability: I selected “No”, though what they have done might be acceptable. The authors don’t provide the (de-identified) documents on which they did content analysis, at least as far as I can discern. It would not be possible to reproduce their work. Perhaps these “raw data” are not required to be shared. Defer to editor.***

It is true that the documents from which the content analyses were done, particularly in the virtual meetings, are not available. The dynamic nature of the real-time Delphi consensus building prevents that. The rationale for not sharing intermediate outputs (ie, data other than what appears in the Supplemental materials) is now explicit in the text (lines 345-346) .

**Other comments:**

***This is an interesting manuscript from which emerges worthwhile messages for improving learning throughout people’s scientific careers. I’m sure the discussions that went into the development of these principles and recommendations for short-format training were fascinating.***

We appreciate this observation and can confirm - the discussions were indeed fascinating!

***Lines 145-164. This is a lot of text to explain essentially what is in the figure. Comes across as repetitive.***

It was not our intention to be repetitive but rather, to ensure that whether the figure or the narrative is more closely attended to, the reader would get the idea of the figure. We also assumed - and this is borne out by the downloads of the preprint in bioRxiv - that the document would be accessed internationally. We opted to ensure that the material is as well-described as possible, rather than save space.

***Line 172-3: Subtle difference between the text and figure here. The figure is, I think, more accurate, by stating "formal knowledge about teaching and learning applies". The authors note in more than one place that the instructors of SFT often don't have pedagogical training (i.e. "formal knowledge about teaching and learning", and as an aside, neither do many, many instructors in FHE). So this formal knowledge is not necessarily a shared characteristic in the real world. But it ought to "apply" to both.***

Exactly; we used "applies", rather than "is used", as the sole overlap between the two types of instruction. However, the language in the figure about "instructors" relates to the expectation, rather than the empirical truth, about preparation. We have inserted the word “ought” to make this clearer as the reviewer suggests.

***Suggestion: perhaps reword slightly to "...the fact that formal knowledge about teaching and learning ought to apply to both forms is perhaps the only shared characteristic"***

The Figure caption has been reworded to capture this (lines 183-184).

***Line 197-99 I think this sentence might be missing a word. "...adopt them"?***

Thank you for catching this, "them" was added (line 220)

***Line 234 (and lots of other places) The 20 items listed in S3 look like a list of questions for discussion – this is not my understanding of the word "vignette". I do recognize that the authors are reporting on meetings they had where they used “vignette”, but it seems like an odd choice.***

We use "vignette" to describe a short and sharp description of a situation, as is often done in work with case analysis. Using "vignette" to describe something considerably shorter than a "case" is common. That is why we chose this label and we've inserted clarifying language to explain what a vignette is in this context. (line 257)

**Line 285-86. *The context stated here that most of the participants are doing this work in countries outside where they are based seems important. Are these folks primarily involved in development work in developing countries (e.g. providing SFTs in topics that are not available locally)? If so, are these settings in which the participants work a relevant aspect of the principles generated? Does this bound the applicability of the principles? Are they particularly suited for this context?***

This is an important point; and part of the rationale for the live/dynamic Delphi was to get real-time feedback on all inputs from participants from experts with all kinds of experience (e.g., work & teach same-country/different countries/many countries). SFT itself is particularly suited to the internationality of life sciences training, " *e.g. providing SFTs in topics that are not available locally)*" — since there might not be local university faculty who can provide technical training in a new method or technique. This might be why the community Principles wheel (sustain, reach, scale) is the 'second' wheel (in meeting discussion, we note that unicycles work and are entirely appropite in our context when training is not meant to be scaled). The Core Principles apply in every training and teaching situation.

***Figure 2. The rear wheel is shown as a "cycle", but it is not clearly explained why these four parts connect in a cycle. In fact, the image shows the four items in a different order than the bullet points below the image. I'd recommend the authors figure out how to better connect the parts of the cycle, if it is one. As a reader, I think these four are more like a good list, though a case could be made for a cycle (e.g. what links "Inclusive" back to "Best Evidence"? Possibly collecting evidence about the inclusivity of a learning experience will contribute to "best evidence"...). The justification for a "cycle" is not communicated yet, in my opinion.***

The cycles were one of four different visualizations we tried with ourselves and with our organizing committee. None of the four elements of the Core Principles "goes ahead" of any other. Instructors may need to focus on just one and the arrows are there to remind instructors and instructional developers that there are other core aspects to be considered. We could not come up with a rationale for a list, a flow diagram or any other configuration so ended with the bicycle. We did switch "catalytic" and "inclusive" in the caption, so the list matches the image.

***For the front wheel, I think the authors are implying an amplifying feedback loop, where if you reach more learners, you need to figure out how to offer more courses or make the courses bigger, or involve more instructors, and, if you want to continue to grow (reach more learners), you need to ensure you have systems in place to support that growth. Reaching more people with effective and inclusive SFTs, and making this possible by sharing good materials and coordinating systems are excellent goals. This wheel is easier to see as a cycle, but what's the end point of this amplifying feedback loop? Saturation of the learner pool?
In general, although I love bicycles, this image seems a bit forced.***

We appreciate the input but again, after weeks of discussion and drawings, we could not defend a rationale for a list, a flow diagram or any other configuration. The bicycle, which represents two cyclic but not necessarily ordered processes won both for its capture of the dynamic process involved and the convenience as a memory aid.

***Line 335: I believe this should reference "S5" instead of "S4.***

Yes thanks for catching that (line 418)

***S6 and Tables 2-5: I recommend that in S6 and in the tables that the authors number the 14 items, rather than use letters. The letters are confusing. E.g. in Table 2, the four recommendations spell FILM. This is a totally unnecessary distraction. Just number these 1-4, then start with 5 in the next grouping. I'm guessing there was much discussion about not wanting to number things which might imply a priority, but lettering simply muddles your list for a reader.
If you don't want to lead with what is currently Recommendation F, lead with L – L (catalysis), to me, is at the heart of this entire effort.***

The group spent a significant amount of time on this very issue (numbering, lettering, or nothing). Since the Recommendation have already been shared globally and have become embedded in multiple projects that we know about, we did not change the labels to numbers or even the ordering of Recommendations. Instead, we simply swapped M and L (so instead of spelling "FILM" the Table1 letters now spell "FIML", which is an acronym for full information maximum likelihood - so, now less likely to be distracting (or, distracting to fewer readers!).

***Table 2: FAIR is used here as an acronym, but these principles are not referenced until line 532.***

The reference was added as a note in Table 2, thanks for catching that (about line 430).

***Lines 352-353. When you say "individuals", do you mean individuals working essentially alone? Or do you mean individual instructors who are part of a larger group of SFT providers who collect evidence from their courses? This sentence implies that funders are most likely to listen to individuals working alone, which might not be what you mean. For example, you note below that it may be difficult for individuals to convince their colleagues to share goals and standards. Funders may be a step harder for individuals.***

This might be a country-specific situation, but an individual instructor might apply for funding to try something new in a course, or, with other instructors. In that situation, the funder (e.g., the US National Science Foundation, or the Swedish Research Council) gets an application with some preliminary evidence from an individual instructor to try something new. One of us has had extensive success convincing funders to support educational research and zero success with the home institution to implement federally the empirical results of federally funded educational research. We meant individuals who are working alone on something, or working as individual instructors at the same or different institutions. Some countries may not have this kind of funding available. We've changed this (line 446) to "individual instructors or instructional developers".

***Reviewer #2: The manuscript by Williams et al. provides a report on the outputs of a project which aimed to improve the delivery and impact of Short Format Training (SFT) workshops. The manuscript describes the process that the project went through to develop the final recommendations that were produced, and then explores the potential implementation of these recommendations across different "user groups" (i.e., individual trainers, communities, institutions).***

***The manuscript is well-written, and does a good job of describing the current state of SFT-based training initiatives (including coverage of current challenges), before moving on to explain the processes that the authors went through to generate the proposed stet of recommendations.

Of note, the authors' highlighting of the relatively low-impact of current SFT (i.e., in terms of how much long-term utility attendees actually gain from this type of training) is extremely valuable (although also rather sobering), particularly in the light of how much has been invested in developing and delivering this style of training in recent years. Rather than dismissing this approach to content delivery, the authors are proposing some useful and readily-implementable recommendations to help improve SFT impact.

Given the popularity and ubiquity of Short Format Training across a wide range of disciplines, this thoughtful and well-structured manuscript is a timely and highly relevant submission.***

We are grateful for these observations!

**Specific comments:**

***1. Line 235 - given the breadth of the potential target audience (i.e., almost anyone involved in developing, delivering and/or coordinating SFT) it might be worth briefly defining "phenomenography", which will not necessarily be something that non-qualitative researchers will be familiar with.***

Thanks for pointing this out, we added a simple explanation and a reference. (lines 261-262)
 ***2. Lines 289-290: it would be useful to see the numbers involved from each country (e.g., "Canada (x), France (y)" etc).***

We did not specify the numbers from each country for several reasons:

1. One person from The Netherlands, Sweden, and Switzerland attended (all of them virtually for these cases) and each of them was the training coordinator for their entire country (so, multiple participants from those countries would not have been better necessarily, just different numbers) whereas several people from the US were really representing totally different segments of the instructor experience and influence;
2. People came from one of the countries in the list but were in fact either from a different country prior to the current job in that country, or, were moving to a different country immediately after the meeting;
3. Some people lived in one country and exclusively taught in different countries. etc.

So the list of countries really reflects the range of attendee experience and expertise, but numbers from each country would not actually be informative in a meaningful way. That is why we list the places, but not numbers "from" each.

***3. Lines 296-298: Presumably the "average" used here was the mean? (this should be specified). Given the highly skewed nature of these figures (e.g., 0-7000 for "new instructors trained"), the median would be a more appropriate measure of the "average". Also, based on the journal's requirements for data availability, the raw data behind these averages needs to be made available (this could easily be done as a small spreadsheet in the supplementary information).***

Such a great point. However, upon checking medians were also not really informative. Instead, we now just give the range, lines 352-354 - which isn't ideal, but some people did not train trainers or engage in SFT at all, or, had done so previously but were now in roles where they did not (thus bringing valuable experience and expertise, as well as newly-influential roles and experience with policy), while others were from organizations that trained thousands but they themselves did not train anyone. A summary like mean or median really doesn't add much, but knowing the upper range and the names of the organizations where participants work (e.g., Carpentries) is probably more informative.

***4. Line 302: "Catalytic learning" should be defined somewhere in the document. I realise that it is loosely defined in Figure 2, but I think a more foraml defintion in the text (or within a specific box - see next comment), along with a relevant citation, would be helpful.***

We added this definition (lines 381-2) with its citation. The citation had been there but not the definition, so this does help to clarify it.

***5. Line 320: as per my comment above about "phenomenography", I think it would be worth briefly defining the Delphi technique here. I wonder if having a "Definitions Box" might be useful, just to help readers who are not coming from an educational research background?***

Delphi method was elaborated for Reviewer 1, now it is also defined with referencees given for interested readers.

***6. Line 342: "i.e.," should be written as "that is" if it appears in the main text (rather than within parentheses).***

Corrected (throughout).

***7. Line 363: it isn't obvious what is meant by "professionalizing" in this context - this should be made clear. It could mean "utilsing a formal paid training system" (which I'm assuming is the intent), but it could also be interpreted as "acting in a more professional manner".***

This is a great point. The recommendation was a focal point for several participants, and we did not have the opportunity to formalize what was meant — but both of the options would probably fit. Given the resistance to actual forward momentum on this topic, it is possible that leaving it undefined/unspecifies is intended to support any movement at all towards a system that is better respected, better paid, and in general has all the attributes of a profession rather than something done in one's spare time which is how teaching, particularly SFT but also teaching at "research" institutions, is too often is treated.

***8. Line 367: the term "formal organizations" is used here without definition. The examples associated with "organizations and institutions" in the next paragraph almost look like a definition for "formal organizations" - could this definition be moved up so that it is clear what "formal organizations" is referring to?***

We defined "formal organizations and institutions" in lines 448-449, and tried to include definitions in this first mention but couldn't without distracting much from the narrative —so, we added "defined below" there at the first mention.

***9. Lines 431-432: reference 50 is talking specifically about ableism, however the authors' mention of "creation of inclusive environments" would generally be taken to refer to more than persons with disabilities (e.g., minorities, neurodiverse individuals, indigenous communities etc). I think the wording in this part of the paragraph needs to be modified to make sure that "inclusive environments" is an broad as possible, and then rephrase the ableist-specific material as one example of how to co-design for inclusivity.***

Great observation. We've modified the language (lines 531-535) to promote the widest meaning of "inclusion" as it underpins recommendation D.
